# Identification of key biomarker genes in liver hepatocellular carcinoma and kidney renal clear cell carcinoma progression: A shared high-throughput screening and molecular docking method with potentials for targeted therapeutic interventions

**DOI:** 10.1016/j.jgeb.2025.100497

**Published:** 2025-04-22

**Authors:** Maisha Tasneem, Shipan Das Gupta, Md Jubair Ahmed Jony, Maya Minkara, Rajib Kumar Dey, Jannatul Ferdoush

**Affiliations:** a Department of Biotechnology and Genetic Engineering, Noakhali Science and Technology University, Noakhali 3814, Bangladesh; b Department of Biology, Geology, and Environmental Science, University of Tennessee at Chattanooga, 615 McCallie Ave, Chattanooga, TN 37403, USA; c Chittagong Medical College Hospital, Chattogram, Bangladesh

**Keywords:** DEGs, LIHC, KIRC, Hub genes, *TOP2A*, Molecular docking, Biomarker

## Abstract

**Background and objectives:**

Liver Hepatocellular Carcinoma (LIHC) and Kidney Renal Clear Cell Carcinoma (KIRC) are leading causes of cancer death worldwide, but their early detections remain hindered by a lack of genetic markers. Our study aims to find prospective biomarkers that could serve as prognostic indicators for efficient drug candidates for KIRC and LIHC treatment.

**Methods:**

To detect differentially expressed genes (DEGs), four datasets were used: GSE66271 and GSE213324 for KIRC, and GSE135631 and GSE202853 for LIHC. Visualization of DEGs was done using heatmaps, volcano plots, and Venn diagrams. Hub genes were identified via PPI analysis and the cytoHubba plugin in Cytoscape. Their expression was evaluated using box plots, stage plots, and survival plots for prognostic assessment via GEPIA2. Molecular docking with PyRx’s AutoDock Vina identified optimal binding interactions between compounds and proteins. Pharmacokinetic and toxicity analyses reinforced the drug-likeness and safety of these compounds.

**Results:**

In this study, 47 DEGs were identified, with the top 10 hub genes being *TOP2A*, *BUB1*, *PTTG1*, *CCNB2*, *NUSAP1*, *KIF20A*, *BIRC5*, *RRM2, NDC80* and *CDC45*, chosen for their high MCC scores. Data mining revealed a correlation between TOP2A expression and clinical survival outcomes in KIRC and LIHC patients. Docking studies of the TOP2A structure identified a promising compound from *Andrographis paniculata* with high binding energy and interactions with TOP2A. Pharmacokinetic and toxicity assessments support its potential as a drug candidate.

**Conclusion:**

Our study emphasizes *TOP2A*’s prognostic significance in KIRC and LIHC and recognizes *Andrographis paniculata* compound as potential therapeutics, offering prospective for enhanced treatment and patient outcomes in these cancers.

## Highlights

• Among 47 common DEGs, 10 hub genes were associated with KIRC and LIHC.
• Overexpression of *TOP2A* stimulates multi-variant cancer progression, significantly associated with KIRC and LIHC progression.
• TOP2A’s molecular docking underscored *Andrographis paniculata* compound as potential therapeutics.

## 1 Introduction

Kidney renal clear cell carcinoma (KIRC), a common subtype of Renal cell carcinoma (RCC), makes up about 75 % of all cases of RCC.^1^, ^2^ RCC is the most prevalent and devastating kidney malignancy in adults which affects the genitourinary system of human.^2^ There are several types of RCC including KIRC, chromophobe (KICH) and kidney renal papillary cell carcinoma (KIRP). Of them, the recovery rate of KIRC is lower than KICH and KIRP.^3^ Importantly, KIRC does not show a positive response to radiotherapy or chemotherapy treatment, however it leads to high mortality rate.^4^ Since KIRC exhibits resistance to radiotherapy and chemotherapy treatment, surgical removal is the only remaining effective treatment for RCC.^5^ Although immunotherapy and targeted therapy options are accessible for treating RCC, the prognosis remains poor for the patients who are not eligible for surgical treatment.^6^ Hence, a holistic treatment approach that encompasses surgical interventions and targeted therapies has been employed to manage KIRC, resulting in significant advancements in enhancing a patient's overall condition.^7^

Recent studies reported that several genes including *HMGCS2*, *DLEU2* were found to be overexpressed in both KIRC and LIHC^8^, ^9^. LIHC, a common form of hepatocellular carcinoma (HCC), stands for the sixth most common form of cancer globally and is accountable for the third highest number of cancer-related deaths all over the world.^10^ Notably, about 80 % of all cases of primary liver cancer are HCC, arising from the liver cells. Research showed that HCC is similar to different types of cancers in its complexity, arises because of the blending effects of genetic changes, cellular environment and environmental influences; all three factors exert influence on growth, development and spread of tumors.^11^

Furthermore, several genes and cellular pathways have been found to be involved in the initiation and progression of HCC. Notably, about 90 % of primary HCC are classified as LIHC as per histopathology subtyping.^12^ Study revealed that the basis for the development of LIHC are hepatitis C or B, alcohol consumption and non-alcoholic fatty liver disease (NAFLD), and metabolic syndrome (MS) and a combination of metabolic disorders.^13^ Moreover, complexity in identifying liver cancer at an early stage has been shown to be one of the reasons for its elevated mortality rate.^14^ Thus, LIHC as well as KIRC are considered as the deadliest forms of cancers because of their hostile nature and modest survival rate. Therefore, understanding the fundamental mechanisms of LIHC and KIRC are critical to identify and characterize biomarkers that can facilitate the diagnosis, treatment, and prediction of prognosis for this disease is an urgent priority. In cancer the significance of biomarker is profound as they play crucial roles in early diagnosis, prognosis and personalizing treatment by predicting patient responses.^15^

**Fig. 1:**
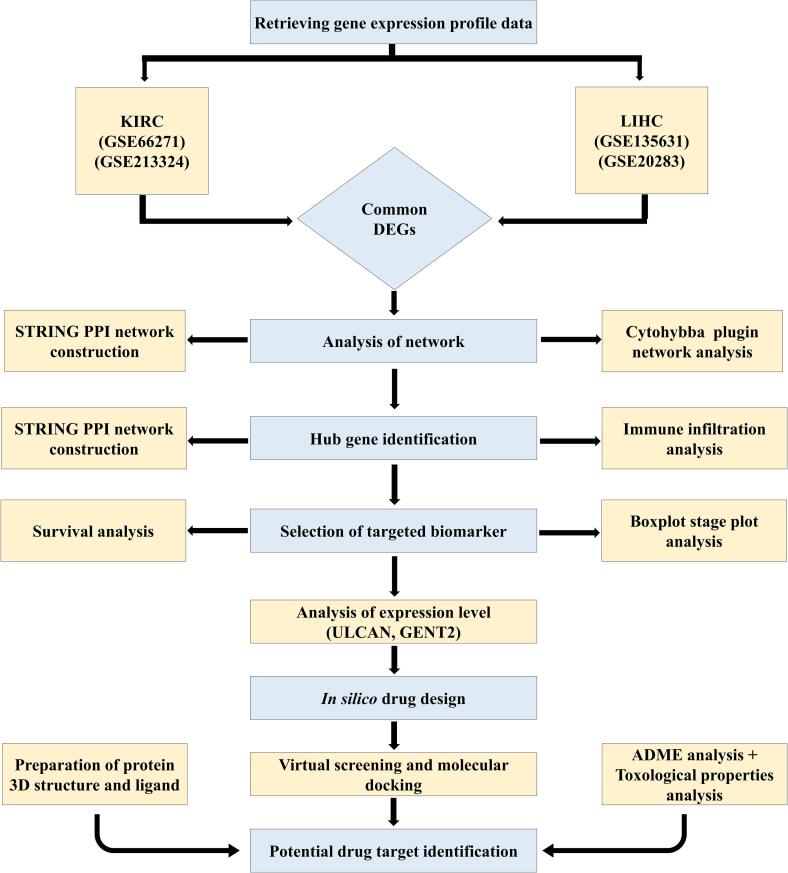
The study's comprehensive procedure.

To achieve this, we analyzed the differential expression of genes (DEGs) in cancerous versus normal tissues using gene expression profiling data from the Gene Expression Omnibus (GEO) and from GEPIA2 database. Briefly, we have selected four gene expression datasets (GSE202853, GSE135631 and GSE66271, GSE213324) to screen for DEGs associated with LIHC and KIRC occurrence and development, respectively. The identification of genes whose expression levels showed notable differences between cancerous and healthy tissues was based on these datasets. A set of bioinformatics analyses was carried out to investigate potential key genes that may be involved in the progression of LIHC and KIRC. Hub genes can play a crucial rule for early cancer detection, diagnosis, and prognosis by reflecting the disease's genetic and molecular changes.^16^ To detect these essential genes, we used protein–protein interaction (PPI) network which helped us to distinguish the associations of these proteins implicated in cancer. Survival analysis was also utilized to evaluate the possible influence of these hub genes on cancer patients' chances of survival. Moreover, we used the CytoHubba plugin's features in the Cytoscape platform to carry out the hub gene expression analysis. This helped us to select genes of particular interest. Importantly, *TOP2A* was identified as a gene of substantial importance throughout our analytical pipeline, exhibiting recurrent occurrences and associations in several aspects of our study. Hence, we chose TOP2A for the molecular docking studies. This study is involved in examining possible connections between TOP2A and phytochemical compounds to identify possible therapeutic applications or modes of action. The entire workflow used to identify DEGs and conduct *in silico* analysis is illustrated in Fig. 1.

## 2 Methods

### 2.1 Dataset collection and specifying parameters

To collect datasets, we used both the GEO and GEPIA2 servers. Gene expression profiles for datasets GSE202853, GSE135631 (for LIHC), and GSE66271, GSE213324 (for KIRC) were obtained from the GEO database (https://www.ncbi.nlm.nih.gov/geo).^17^ In GEPIA2 (https://gepia.cancer-pku.cn), we searched for ‘kidney renal cell carcinoma’ and ‘liver hepatocellular carcinoma’ datasets using these keywords.^18^ GEPIA used a differential analysis approach called LIMMA with precise conditions: a log2FC (logarithm of fold change) cutoff value of 1.5 and a q-value cutoff of 0.01.

### 2.2 **Data processing of differential expressed** genes **(DEGs)**

NCBI (https://www.ncbi.nlm.nih.gov/ geo/geo2r/)^17^ GEO2R analysis tool was employed to select the differentially expressed genes (DEGs) between the tumor and normal tissues in the datasets. For each dataset, a volcano plot was created using the DEGs with the Volcano Plot online server (https://paolo.shinyapps.io/ShinyVolcanoPlot/),^19^ which is presented on shinyapps.io by RStudio. After that, the subsequent datasets of DEGs were used for downstream analysis. Overlapping parts of both datasets were selected using the Venn diagram online tool, Venny (https://bioinfogp.cnb.csic.es/tools/venny/). Overlapping genes between the datasets were considered as common DEGs between the datasets.

**Construction of protein**–**protein interaction (PPI) network to identify hub genes:** To visualize the protein–protein interaction (PPI) network, Cytoscape (https://cytoscape.org/; version 3.9.1)^20^ was used. It is created by the Search Tool for the Retrieval of Interacting Genes (STRING) version 11.5, a regularly used software system for predicting and analyzing protein–protein interactions.^21^ We also incorporated an extra plugin called “cytohubba”^20^ to isolate top hub genes in our protein–protein interaction (PPI) analysis.

### 2.3 Functional annotation of targeted DEGs via GO and pathway analysis using Enrichr

Since DEGs are involved in regulating the expression of their targeted genes, therefore it is important to annotate the biological function of the targeted DEGs. Hence, we executed the gene ontology (GO) and pathway analysis by using Enrichr web server ((https://maayanlab.cloud/Enrichr/).^22^

### 2.4 **Immune infiltration** analysis **of hub genes in KIRC and LIHC using TIMER**

Tumor Immune Estimation Resource (TIMER, https://cistrome.shinyapps.io/timer/)^23^ is an extensive web resource for the methodical assessment of immune infiltrates across a range of cancer types, based on the microarray expression values. By using TIMER, the immune infiltration estimation of the hub genes was performed in KIRC and LIHC datasets.

### 2.5 Survival analysis and validation of identified hub genes

The survival analysis of DEGs was performed using the GEPIA2 server.^18^ Given a list of customizable cancer types, GEPIA2 would produce a graph to show the survival analysis result based on multiple cancer types. To use the GEPIA 2 web portal to examine the prognostic significance of the top targeted DEG, differential expression analysis, patient survival analysis, and stage plot analysis.^18^ Additionally, to analyze the expression pattern of the preferred key DEG in the major tumor stages with TPM scale, UALCAN^24^ web portal was employed. Moreover, for stage plot analysis, P-value ≤ 0.05 was considered as statistically significant. Dataset was delimited to KIRC and LIHC in all of the above-mentioned analysis. Finally, DEG’s expression profile in kidney and liver tissues was investigated using GENT2,^25^ and a two-sample T-test was executed.

### 2.6 Identification of transcription factor (TF)-gene interaction

Using Network Analyst v3.0 (https://www.networkanalyst.ca/NetworkAnalyst/home.xhtml),^26^ we found the transcription factors (TFs) that control the top identified DEG’s expression level.

### 2.7 Preparation of protein 3D structure and Ligand

To identify the 3-D experimental tertiary structures of the human TOP2A protein particularly the structure with the PDB ID: 1ZXM, the RCSB Protein Data Bank (https://www.rcsb.org/)^27^ website was employed. Once the protein structure from the PDB was retrieved, downstream preparations and modifications were performed using Discovery Studio 2021, available at (https://www.3ds.com/productsservices/biovia/products/molecular-modeling simulation/biovia-discovery-studio/).^28^ Next, we conducted the energy using the GROMOS96 force-field, which is available within the SWISS PDB Viewer tool, accessible at (https://spdbv.vital-it.ch/).^29^

A complete scientific literature review was executed to find several bioactive phytochemicals from plants. This painstaking investigation produced a total of 77 phytochemical compounds, which were subsequently found in the Structure-Data File (SDF) format from PubChem^30^. To analyze these compounds further, an energy minimization process was used. This scheme uses the Universal Force Field (UFF) and the Conjugate Gradients algorithm, via integrating into PyRx.^69^ All these methods were used to optimize the ligands' structures, confirming that they were in a stable and energetically favorable conformation. This step is essential for consequent computational analyses and simulations linked to these bioactive phytochemicals.

### 2.8 **Blind molecular docking and virtual** screening

To execute molecular docking, we used AutoDock4 and AutoDockVina, the two most effective tools within PyRx. We also used BIOVIA visualizer in Discovery Studio 2021 to see the binding orientation of the protein–ligand complex.

### 2.9 **Analysis of toxicological** properties **and absorption, distribution, metabolism, excretion (ADME)**

To predict several PK properties and analyze the drug-likeness of small molecules, a powerful and accessible tool called SwissADME was used, which is available via the website (https://www.swissadme.ch).^31^ We used two online tools, i.e. the admetSAR 1.0 web-server^32^ and the Protox-II online server (II/index. php)^33^ to test the toxicity levels of the selected compounds.

## 3 Results

### 3.1 Gene Expression Profiling and DEGs Analysis in KIRC and LIHC using GEO Datasets identified 736 KIRC genes, 219 LIHC genes and 47 common genes

**Fig. 2:**
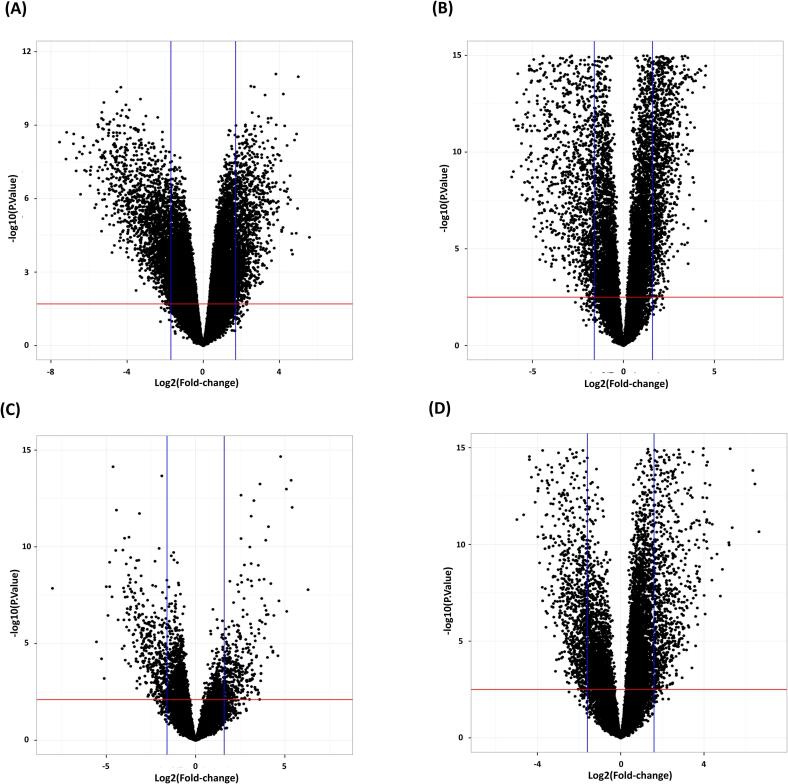
Volcano plots for DEGs in KIRC according to the datasets (A) GSE66271 (B) GSE213324 and LIHC according to the datasets (C) GSE202853 (D) GSE135631.

**Fig. 3:**
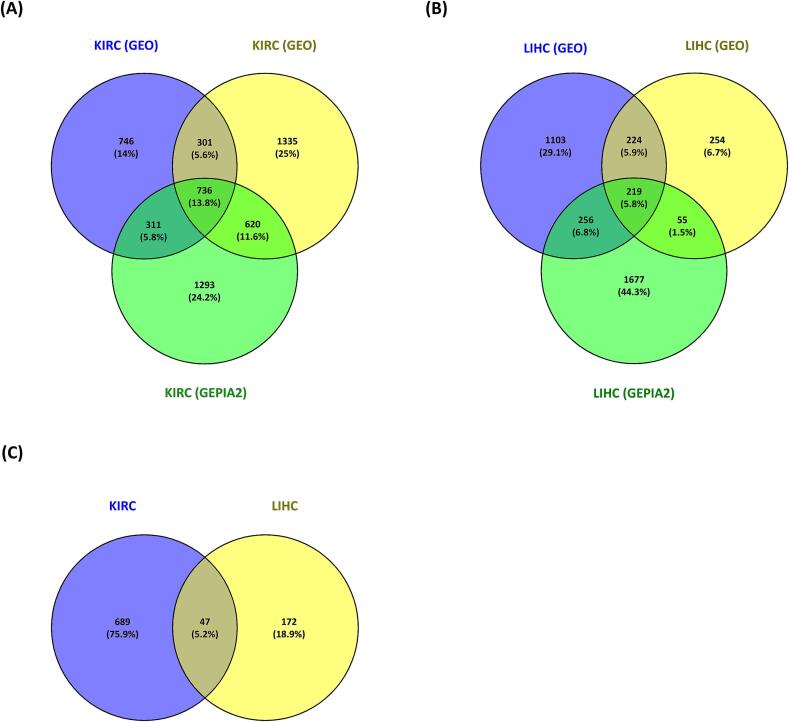
Desired differentially expressed genes (DEGs) with overlap found in KIRC and LIHC. Overlapping DEGs for (A) the GEO and GEPIA2 dataset for KIRC (B) the GEO and GEPIA2 dataset for LIHC (C) the final shared dataset from both cancers are indicated by the numbers in the shared intersecting region.

This study investigated gene expression profiles in Kidney renal cell carcinoma (KIRC) and Hepatocellular carcinoma (LIHC) using four GEO datasets: GSE66271, GSE213324 for KIRC, and GSE135631, GSE202853 for LIHC. KIRC datasets comprised 26 and 41 samples, while LIHC datasets contained 30 and 20 samples, respectively (Table 1). DEG analysis identified 2962 genes in KIRC and 2208 genes in LIHC. Volcano plots were generated with thresholds of P < 0.05 and |log2FC| > 1.5 (Fig. 2). A combined analysis using Venn diagrams identified 736 KIRC genes and 219 LIHC genes, with 47 common genes selected for further investigation (Fig. 3).

**Table 1:** Details on the four GEO datasets used in this study.

**Datasets**	**Number of Samples (tumor/control)**
GSE213324	**41** (20/21)
GSE66271	**26** (13/13)
GSE202853	**20** (10/10)
GSE135631	**30** (15/15)

### 3.2 **Constructing a** protein–**protein interaction (PPI) network reveals the molecular insights of DEGs**

**Fig. 4:**
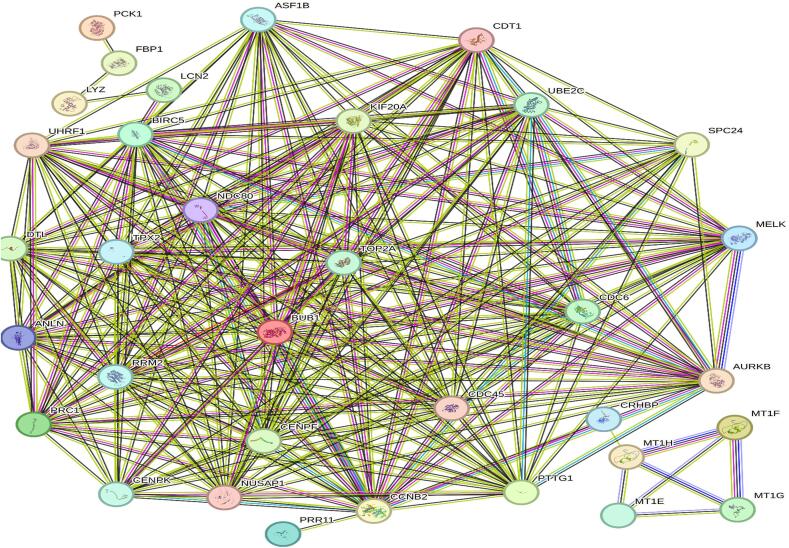
PPI network construction via STRING. The common DEGs of the KIRC and LIHC datasets interact with one another as seen in the network. The initial shell of interactors and query proteins are colored nodes. White nodes: the second layer of interaction agents. Proteins with an unknown three-dimensional structure are called empty nodes. Filled nodes: An estimated or known 3D structure exists. Network nodes: Proteins are represented by these nodes. Protein associations are represented by edges.

Protein-protein interaction analysis of 47 genes was performed using the STRING 12 database. The resulting network comprised 46 nodes and 326 edges, with an average node degree of 14.2. Significant PPI enrichment observed (p < 1.0e-16), with average local clustering coefficient of 0.699 (Table 2). PPI network exported as txt, then transformed to csv for visualization in Cytoscape (Fig. 4).

**Table 2:** Network status of common DEGs.

Number of edges:	276
Number of nodes	34
Average node degree	16.2
Expected number of edges	19
Avg. local clustering coefficient	0.835
PPI enrichment p-value	< 1.0e-16

### 3.3 **Identification of hub genes via PPI network** analysis

**Supplementary Figure 1:**
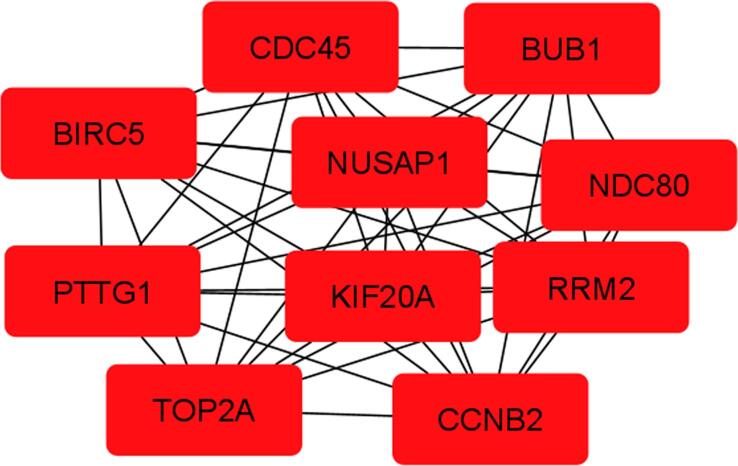


Following examining STRING tool results, we found significant genes within the network by evaluating nodes with higher degree, closeness, and betweenness scores in comparison with the mean estimated by cytoHubba. We isolated the top 10 hub genes (*TOP2A*, *CCNB2, BUB1, NUSAP1*, *KIF20A*, *NDC80*, *BIRC5*, *RRM2* and *CDC45*) from the PPI network by using the MCC algorithm (Supplementary Fig. 1). The different degrees of significance of these genes were indicated by color coding, which ran from yellow to red. Cytscape software, which was obtained from the STRING database, was used to process the uploaded.tsv file. Next, using Cytohubba's gene selection process, a cluster of 10 closely related genes expressed in both KIRC and LIHC was found (Table 3).

**Table 3:** Using the MCC method, the top 10 networks.

**Rank**	**Name**	**Score**
1	*TOP2A*	1.18E + 21
2	*PTTG1*	1.18E + 21
3	*BUB1*	1.18E + 21
4	*CCNB2*	1.18E + 21
5	*KIF20A*	1.18E + 21
6	*NUSAP1*	1.18E + 21
7	*BIRC5*	1.18E + 21
8	*NDC80*	1.18E + 21
9	*RRM2*	1.18E + 21
10	*CDC45*	1.18E + 21

### 3.4 **Functional enrichment analysis uncovers a range of biological signatures associated with hub genes in KIRC** and **LIHC**

**Fig. 5:**
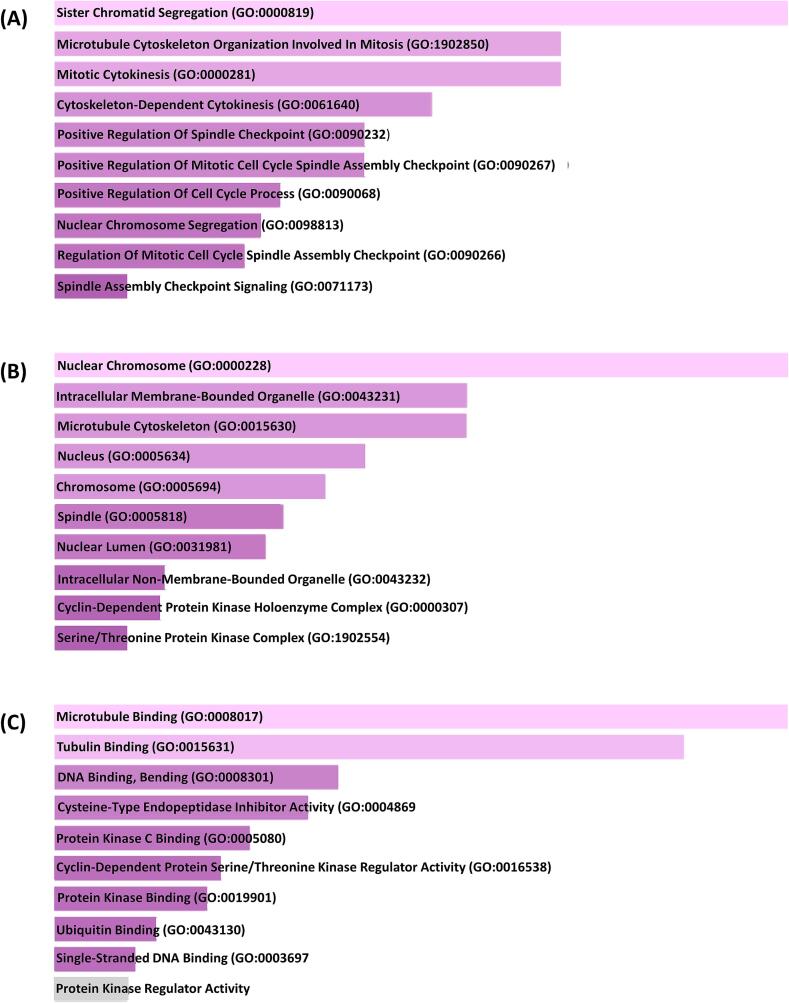
The 10 differentially expressed genes (DEGs) in KIRC and LIHC showed a significantly enriched Gene Ontology (GO) functional enrichment study. The terms that rank highest among biological processes (A), cellular components (B), and molecular functions (C) are listed.

The chosen hub genes were entered into the Enrichr online analysis tool in order to discover annotation, possible Gene Ontology (GO) classifications, and enriched KEGG pathways within the dataset. The Biological Process enrichment analysis disclosed that the DEGs demonstrated associations with numerous processes including the microtubule cytoskeleton organization involved in mitosis, the mitotic spindle arrangement, sister chromatids separation during mitosis, mytotic cytokinesis, mitotic spindle assembly checkpoint positive regulation and the cell cycle process regulation, separation of nuclear chromosome, etc. (Fig. 5-A). The downstream investigation of cellular component showed that the DEGs exhibited associations with nuclear chromosome, intracellular membrane bounded organelle, cytoskeleton (microtubule), spindle, nucleus, nuclear lumen, chromosome, intracellular non-membrane bounded organelle, serine/threionine protein kinase complex (Fig. 5-B).

In terms of molecular function, the DEGs were predominantly enriched in activities such as tubulin binding, microtubule binding, interactions with protein kinases, ubiquitin binding, DNA binding and bending, single stand DNA binding, and protein kinase regulatory activity. (Fig. 5-C).

**Supplementary Figure 2:**
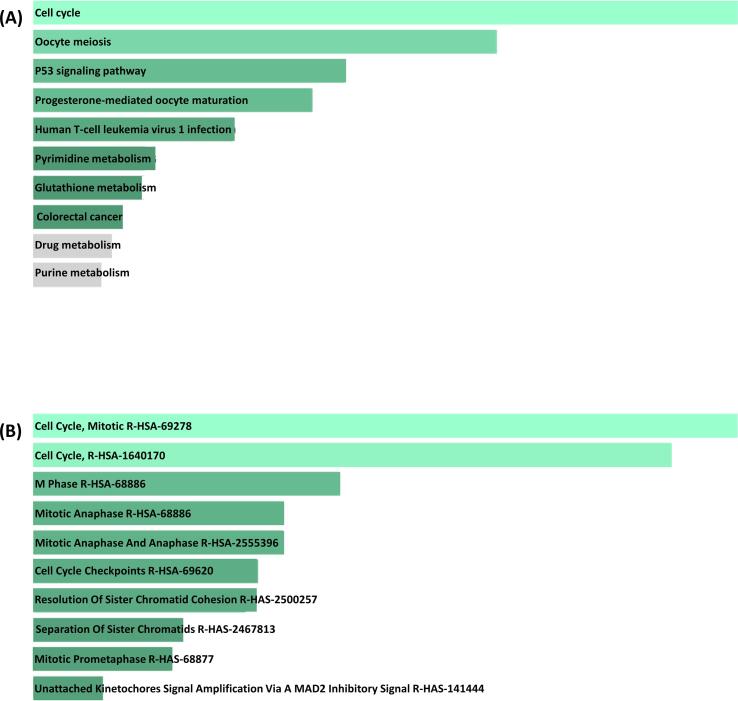


To study the essential pathological pathways connected to the ten chosen DEGs during the emergence and progression of KIRC and LIHC, Enrichr was used. The top ten important KEGG pathways were assessed based on their P-values (<0.05). The KEGG pathway investigation showed that the ten selected DEGs were considerably associated with several pathways, including the p53 signaling pathway, the cell cycle regulatory pathway, progesterone mediation facilitated oocytes maturation, meiosis of oocyte, metabolism of pyrimidine, metabolism of glutathione, and colorectal cancer (Supplementary figure 2-A). Reactome pathway results indicated that 10 targeted DEGs had a substantial role in cell cycle, cell cycle checkpoints, mitotic prometaphase, metaphase and anaphase etc. (Supplementary figure 2-B).

### 3.5 **Exploring Gene Correlations with Tumor** Purity **and Immune Cell Abundance in KIRC and LIHC Using TIMER**

**Supplementary Figure 3:**
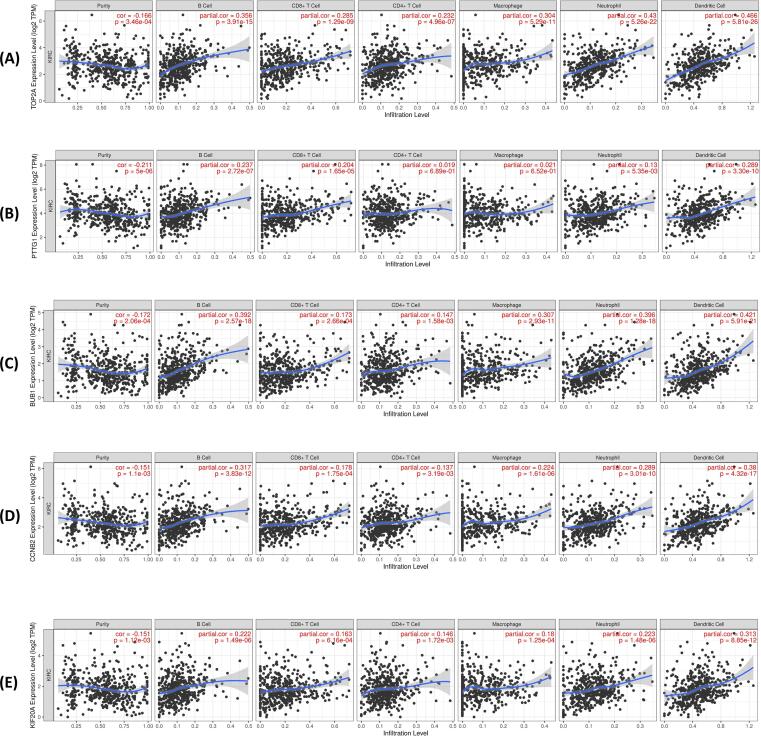


**Supplementary Figure 4:**
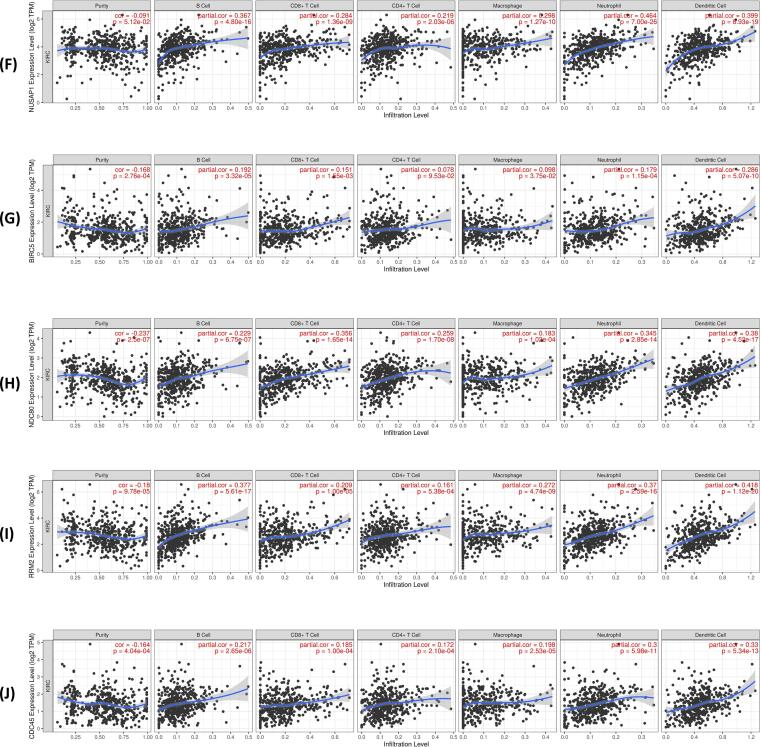


**Supplementary Figure 5:**
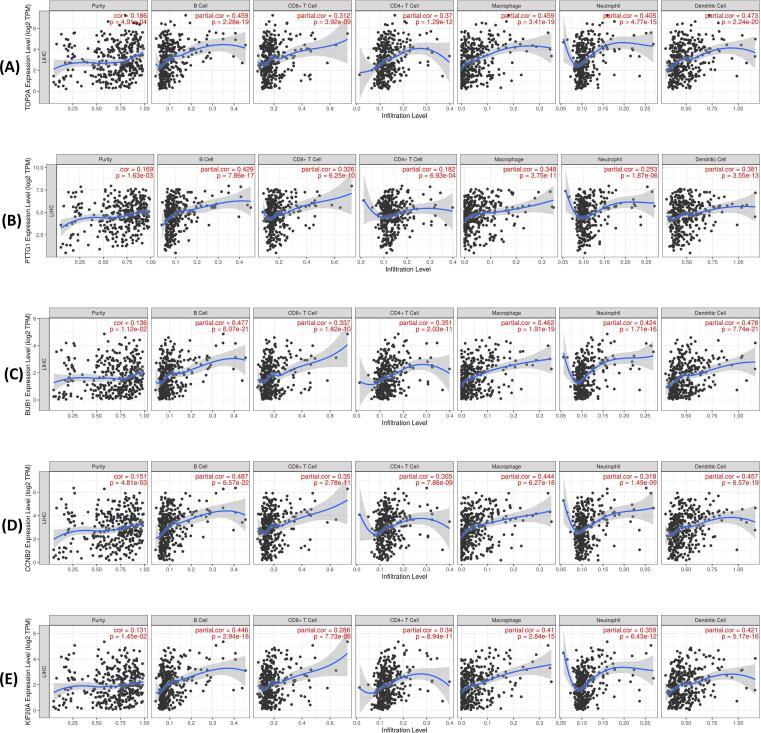


**Supplementary Figure 6:**
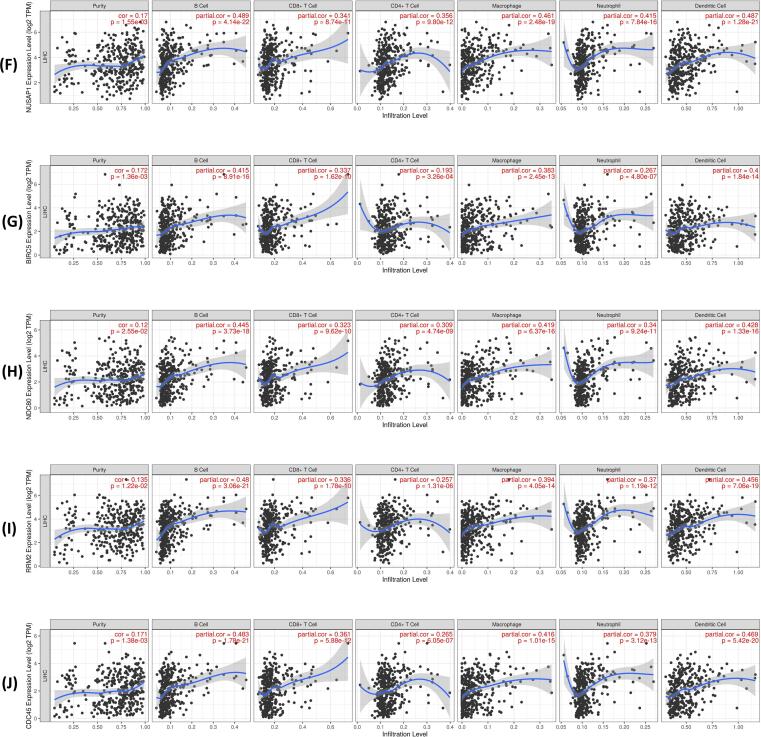


Our investigation using the TIMER tool uncovered intriguing correlations in KIRC and LIHC. In KIRC, genes like *TOP2A*, *PTTG1*, and others showed a negative link with tumor purity but a positive association with various immune cell types. Similarly, in LIHC, the top 10 hub genes exhibited strong positive correlations with both tumor purity and immune cell abundance. These findings underscore the potential of our prognostic model in predicting cancer prognosis effectively (Supplementary Fig. 3-6).

### 3.6 Selection of *TOP2A* as a Key Biomarker in KIRC and LIHC using CytoHubba Analysis

**Supplementary Figure 7:**
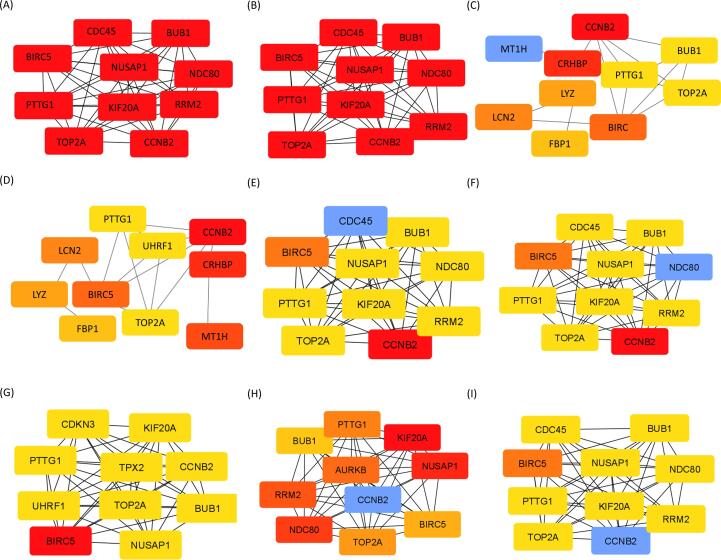


The cytoHubba plugin in Cytoscape was employed to analyze 10 hub genes assessing their significance through various ranking techniques including degree, bottleneck, and clustering coefficient. Among these genes, *TOP2A* emerged as the most prominent candidate, consistently identified across all nine cytoHubba methods employed. Additionally, *KIF20A* was detected in six methods, albeit less consistently than *TOP2A*. Given *TOP2A*'s consistent performance and prior computational relevance to KIRC and LIHC, it was selected for further investigation through docking studies, supported by a visual representation illustrating its prevalence across the employed cytoHubba methods (Supplementary Fig. 7).

### 3.7 Prognostic analysis of *TOP2A* based on patients pathological and survival analysis

**Fig. 6:**
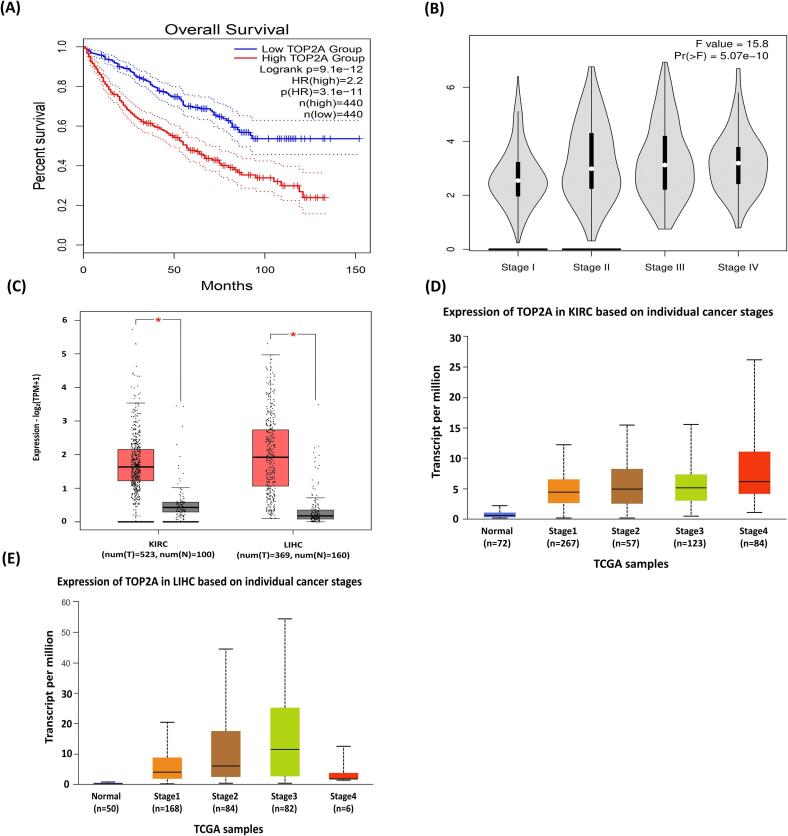
Prognostic analysis of TOP2A in KIRC & LIHC: (A) Survival Analysis; Overall survival analysis of patients with KIRC & LIHC. (B) Stage plot analysis; Expression of TOP2A in individual cancer stage (stage I, stage II, stage III, and stage IV) in both KIRC & LIHC. (C) Box Plot analysis; Expression profile of TOP2A (Tumor (red) Normal (ash)) of TOP2A in KIRC & LIHC. (D, E) ULCAN analysis: Expression of TOP2A in different tumor stages. (For interpretation of the references to color in this figure legend, the reader is referred to the web version of this article.)

In GEPIA2 based survival analysis, the correlation between gene expression and patient prognosis was investigated for *TOP2A*. *TOP2A* gene demonstrates the greatest association with survival outcome (Fig. 6-A). A stage plot analysis was then conducted, considering both F and P values to rank genes based on differential expression across stages. *TOP2A* emerged with the highest F value, indicating significant expression according to both F and P values (Fig. 6-B). Boxplot investigations confirmed markedly higher expression levels of *TOP2A* in KIRC and LIHC patients compared to normal individuals, suggesting their overexpression in these conditions (Fig. 6-C). Furthermore, GENT2 utilized to assess gene expression in liver and kidney cancer tissues where *TOP2A* showed significant upregulation in both cancers, with high statistical significance and fold change values (Table 4). Further validation using UALCAN and TCGA data also confirmed elevated expression *TOP2A* across cancer stages and grades, indicating their crucial involvement in kidney and liver cancers (Fig. 6-D&E).

**Table 4:** Evaluate expression levels of 4 genes by GENT2 analysis.

**Gene**	**Tissue**	**P- value**	**Log2Fc**
*TOP2A*	Kidney	<0.001	2.480
	Liver	<0.001	2.607
*PTTG1*	Kidney	<0.001	1.387
	Liver	<0.001	1.286
*KIF20A*	Kidney	<0.001	2.520
	Liver	<0.001	2.884
*BUB1*	Kidney	<0.001	1.188
	Liver	<0.001	1.974

### 3.8 Transcription factors linked to the regulation of *TOP2A* expression

**Supplementary Figure 8:**
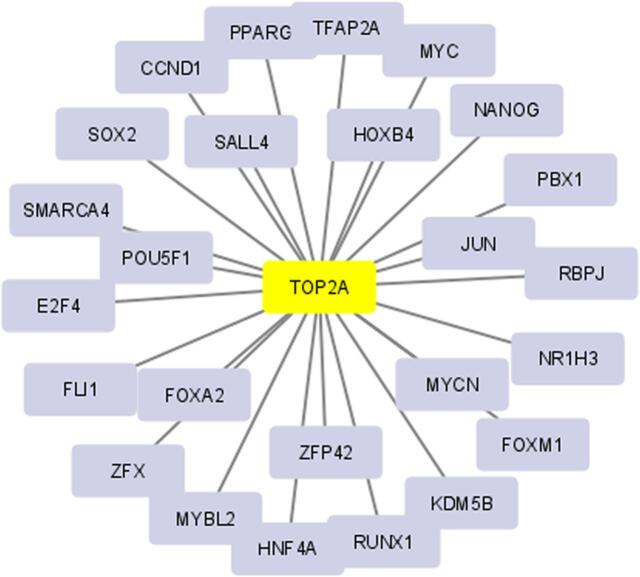


Network Analyst v3.0 was employed to find the transcription factors that exhibit critical role in regulating the expression of the *TOP2A* gene. The interaction network elucidates the associations of these transcription factors with *TOP2A*, with 25 nodes depicting the factors, 24 edges showing their connections, and 1 seed as the starting point. The network contains a total of 25 DNA-binding factors that precisely bind to the *TOP2A* gene. These 25 transcription factors, including CCND1, TFAP2A, MYC, *NANOG*, PPARG, PBX1, RBPJ, FOXM1, KDM3B, ZFP42, UNF4A, MYBL2, ZFX, JUN, FLIX, FOXA2, E2F4, POUF1, NR1H3, SMARCA4, SOX2, RUNX1, MYCN, and SALI4, could coordinate *TOP2A* expression by either stimulating or supressing transcription (Supplementary figure 8).

### 3.9 **Molecular docking studies** with ***TOP2A* identified potential therapeutic compounds**

**Fig. 7:**
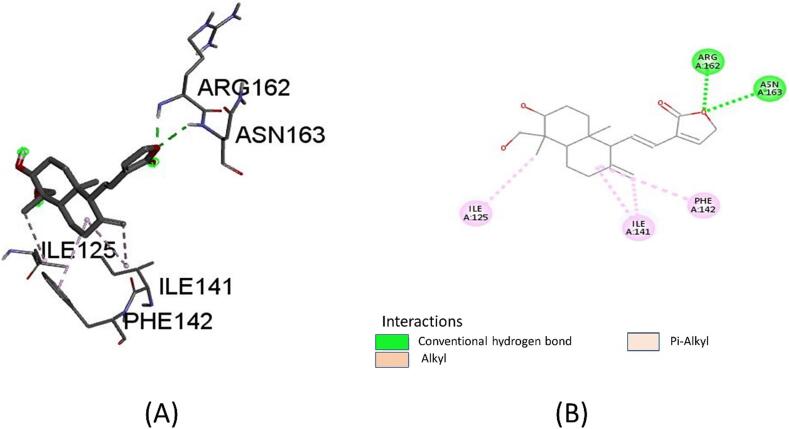
TOP2A's protein ligand interaction with Human Topo IIa ATPase/AMP-PNP. The two conventional hydrogen bonds that 14-Deoxy-11,12-didehydroandrographolide formed with ARG (162) and ASN (163) were demonstrated in (A) and (B). Additionally, it interacted with ILE (125) Ile (141) ILE (141) forming both an alkyl bond and a Pi alkyl bond with PHE (142).

The docking study utilized the crystal structure of TOP2A bound with Human Topo IIa ATPase/AMP-PNP (PDB ID: 1IZXM). Docking was performed using the Natural Compound Library sourced from the Indian Medicinal Plants, Phytochemistry, and Therapeutics (IMPPAT) database. The binding energies of the compounds were assessed, with the standard inhibitor demonstrating a binding energy of −7.0 kcal/mol. From the results, one compound was selected based on their maximum binding energies: 14-Deoxy-11,12-didehydroandrographolide with docking scores of −9.5 kcal/mol. This compound was further analyzed for its interactions with the protein which formed conventional hydrogen bonds with ARG (162) and ASN (163), along with alkyl and Pi-alkyl bonds with other residues (Fig. 7). This finding provides valuable insights into the potential mechanisms of action of this compound as inhibitors of Human Topo IIa ATPase/AMP-PNP, highlighting their potential for further investigation in drug discovery endeavors.

### 3.10 **Prediction of ADME** properties **and toxicity for selected therapeutic compounds**

**Fig. 8:**
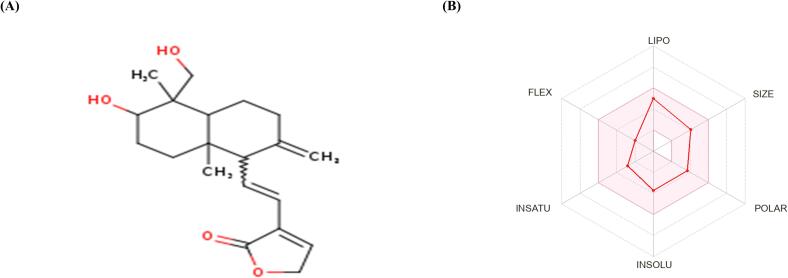
Features of 14-Deoxy-11,12-didehydroandrographolide according to ADME: A suitable range of physio-chemical properties for oral bioavailability is represented by the shaded area in which 14-Deoxy-11,12-didehydroandrographolide falls; (B) Specific properties like polarity (POLAR), insolubility (INSOLU), instauration (INSATU), rotable bond flexibility (FLEX), lipophilicity (LIPO), and molecular weight (SIZE) are displayed.

In our study, we analyzed 14-Deoxy-11,12-didehydroandrographolide using Canonical SMILES notation on the SwissADME platform. The drug-likeness graph generated depicted peaks representing parameters that specify different drug qualities. The delineated pink region on the chart indicates the ideal range for every attribute, with compound falling within this zone considered potential candidate (Fig. 8). We predicted the toxic traits of our chosen candidate using the admetSAR server. The analysis revealed no indication of AMES toxicity for this selected compound (Table 5). To validate these results, we corroborated the toxicity data using the ProTox-II online server for the specified compound (Table 6).

**Table 5:** Toxicological properties of 14-Deoxy-11,12-didehydroandrographolide include drug-induced hERG inhibition, AMES toxicity, carcinogens, and Acute oral toxicity.

**Compound (Pubchem ID)**	**hERG inhibition**	**AMES**	**Carcinogens**	**Acute oral toxicity**	**Carcinogenicity**
5,708,351	Weak	No	No	III	Not required

**Table 6:** Toxicity profiling of 14-Deoxy-11,12-didehydroandrographolide through Protox-II Online server.

**Compound (Pubchem ID)**	**Mutagenicity**	**Immunogenicity**	**Cytotoxicity**	**Carcinogenicity**
5,708,351	Inactive	Inactive	Inactive	Inactive

## 4 Discussion and future prospect

LIHC and KIRC are aggressive cancers with high mortality rates due to their rapid metastasis, resistance to treatment, and poor prognosis. Despite recent research efforts, early diagnosis, effective treatment, and favorable outcomes for these cancers remain elusive. Consequently, it is imperative that we expand our comprehension of the molecular mechanisms involved in the development and progression of LIHC and KIRC to identify potential targets for diagnosis and therapy.

In this study, 47 differentially expressed genes (DEGs) were identified, with the top 10 hub genes being *TOP2A, RRM2, CCNB2, PTTG1, NUSAP1, KIF20A, BIRC5, NDC80, BUB1,* and *CDC45*, chosen for their high MCC scores. A correlation between *TOP2A* expression and clinical survival outcomes in patients with KIRC and LIHC was revealed through data mining. Protein-protein interaction (PPI) network analysis revealed significant molecular interactions among these DEGs, highlighting their potential roles in cancer progression. Functional enrichment analysis indicated that these hub genes are involved in critical biological processes, including cell cycle regulation, mitosis, and spindle assembly. The TIMER tool showed that these genes are correlated with immune cell infiltration and tumor purity in both KIRC and LIHC, suggesting their relevance in the tumor microenvironment. Notably, *TOP2A* emerged as a key biomarker, consistently associated with poor survival outcomes in patients with KIRC and LIHC, as confirmed through survival and stage plot analyses. Elevated expression of *TOP2A* was further validated in cancer tissues using GENT2 and UALCAN databases. Given its consistent upregulation and significant correlation with patient prognosis, *TOP2A* was selected for molecular docking studies. The docking results identified 14-Deoxy-11,12-didehydroandrographolide as a potential therapeutic compound, demonstrating strong binding affinity with *TOP2A*. The compound, 14-Deoxy-11,12-didehydroandrographolide is derived from the common herb *Andrographis paniculata*. ADME and toxicity predictions for this compound indicated favorable drug-likeness properties, positioning it as a potential candidate for additional investigation in cancer treatment.

Since our study revealed that *TOP2A* overexpression is significantly associated with the progression of LIHC and KIRC, it is crucial to explore the gene's critical function in developing potential therapeutics. *TOP2A* (DNA Topoisomerase II Alpha) encodes a DNA topoisomerase, an enzyme that regulates the topologic states of DNA during replication and transcription. It is also implicated in regulation of several cellular processes such as chromosome condensation, chromatid separation, and the relief of torsional stress that happens during DNA transcription and replication. A recent study showed that LIHC tissues had substantially higher *TOP2A* gene expression levels than did normal tissues. Additionally, comparing the clinical stage and gene expression revealed that *TOP2A* expression rose as the tumor reached more advanced stages. According to methylation analysis, tumor tissue had substantially less methylation of the *TOP2A* gene than normal tissue. This suggests that *TOP2A* expression and methylation status may be important factors in tumor development and progression. Therefore, the overexpression of *TOP2A* can be utilized as a LIHC prognostic indicator.^34^ Moreover, inhibiting *TOP2A* gene was found to significantly reduce growth and mobility of KIRC cells. These findings suggest that programmed cell deaths are important in predicting outcomes and responses to immunotherapy in KIRC, offering insights that could improve treatment approaches for KIRC.^35^

Another important hub gene, Pituitary tumor-transforming gene-1 (*PTTG1*) encodes a global transcription factor which exhibited many transcriptional targets that are implicated in diverse cellular processes. For instance, *PTTG1* activates c-Myc and thus play a role in cell transformation. *PTTG1* also activates fibroblast growth factor 2 expression and stimulates tumor angiogenesis. Moreover, it impedes p53 transcriptional activity. It also represses p21 expression and activates cyclin D3, suggesting a function in cell cycle regulation and senescence.^36^ Importantly, a recent study determined that KIRC tissues had significantly higher levels of *PTTG1* expression than normal tissues. Regression analysis revealed that high *PTTG1* expression was an independent predictor of overall survival, correlating with shorter survival in KIRC patients. Gene set enrichment analysis uncovered seven *PTTG1*-related pathways, and *PTTG1* was strongly associated with both the immune system and tumor mutational burden (TMB) in KIRC. Patients with lower *PTTG1* expression responded better to immunotherapy, suggesting that *PTTG1* is closely linked to TMB, immunity, and prognosis in KIRC patients.^37^ Moreover, another study showed that *PTTG1* function was suppressed by a drug called Falcarindiol (FAD). FAD slow down cell growth, weaken DNA repair, and increase apoptosis, making LIHC cells more responsive to the chemotherapy drug cisplatin (DDP). FAD and DDP worked synergistically to suppress cancer cell growth more effectively, and therefore could be used as a promising cancer treatment that can enhance chemotherapy effectiveness in liver cancer by targeting the *PTTG1* pathway.^38^

The hub genes, *BUB1, KIF20A* and *NUSAP1* play important roles in mitosis. *BUB1* is a conserved serine/threonine kinase which has been found to suppress anaphase promoting complex/cyclosome (APC/C), thus play a role in DNA damage response during mitosis.^39^ Therefore, mutations in *BUB1* have been linked to many cancers. Essentially, a group of researchers performed immunohistochemistry and found elevated *BUB1* levels in cancerous tissues. They also showed that reducing *BUB1* expression decreased LIHC cell proliferation, migration, and signaling proteins while showing a connection to immune cell infiltration. Therefore, *BUB1* may serve as both a prognostic biomarker and an indicator of immune status in LIHC.^40^ Similarly, another study demonstrated that *BUB1B* expression in KIRC was linked to higher nuclear grade, T stage, M stage, and poor overall survival. Immunohistochemistry revealed that *BUB1B* expression was associated with markers like CD44, p53, and PD-L1, with knockdown experiments showing reduced KIRC cell growth and invasion upon *BUB1B* depletion. This study concludes that *BUB1B* may serve as both an oncogenic factor and a predictive biomarker for KIRC survival.^41^ *KIF20A* (Kinesin family member 20A), a cytokinesis regulator, transports chromosomes during mitosis. Thus, regulation of *KIF20A* expression is important for normal cellular function. Importantly, *KIF20A* was found overexpressed in several cancers including LIHC and KIRC.^42^ Experimental studies revealed that knocking down *KIF20A* suppressed LIHC cell proliferation and increased chemosensitivity to cisplatin and sorafenib, suggesting that *KIF20A* exhibits critical function in LIHC progression and could be used as potential target for future therapeutic interventions.^43^ The hub gene, *NUSAP1* (Nucleolar and Spindle-Associated Protein 1) is a critical mitotic regulator which is involved in regulation of mitotic microtubule stability and chromosome segregation. Importantly, *NUSAP1* acts as an oncogene implicated in numerous cancers’ progression. It is also found overexpressed in several cancers including LIHC and KIRC. A wet lab study found that Silencing *NUSAP1* hindered cell proliferation and stimulated apoptosis of cell. ^44^ NUSAP1 levels were measured using qPCR and western blotting in LIHC and KIRC tissues and adjacent normal tissues, revealing significant upregulation in LIHC and KIRC samples and associations with poorer patient outcomes. Functional assays demonstrated that elevated *NUSAP1* levels enhance LIHC and KIRC cell proliferation and tumorigenicity, promoting cell cycle progression. These findings suggest that *NUSAP1* plays a critical role in LIHC and KIRC development and could be a valuable prognostic marker^45^, ^46^.

The other hub genes, *CCNB2, CDC45* and *NDC80* are cell cycle regulator genes. Cyclin B2 (*CCNB2*) is a B-type cyclins which plays a critical role in G2/M checkpoint. Abnormal *CCNB2* expression may lead to the failure of G2/M checkpoint and may activate gene mutations and tumorigenesis. Notably, *CCNB2* was found abnormally expressed in various types of cancers, including LIHC and KIRC. *CCNB2* expression levels increased with tumor grade and stage, correlating with poor clinical outcomes such as metastasis, recurrence, chemotherapy resistance, and decreased overall survival. These results suggest that *CCNB2* is crucial for maintaining LIHC stemness.^47^ Another study identified a “green module” of genes highly associated with KIRC clinical outcomes, particularly enriched in pathways related to mitotic nuclear division, the cell cycle, and the p53 signaling pathway. Among the 26 candidate genes, *CCNB2* was included in a 9-gene prognostic model with strong predictive ability for KIRC prognosis, with all nine hub genes generally overexpressed in KIRC tissues. Thus, *CCNB2* and these other genes are critical for understanding KIRC progression and could be valuable for tailoring treatments.^48^ The Cell division cycle protein 45 (*CDC45*) is essential for DNA synthesis during cell division. *CDC45* partners with the replisome CMG helicase (minichromosome maintenance proteins (Mcm2–7)) to support the motor activity for uncoiling the DNA during replication.^49^ Notably, recent studies showed that high *CDC45* expression correlates with shorter survival in several cancer patients including LIHC and KIRC patients. Experiments involving the manipulation of *CDC45* levels in LIHC cells showed that overexpression of *CDC45* enhances cell viability and proliferation through the ERK1/2 signaling pathway, while inhibiting apoptosis. Silencing *CDC45* had the opposite effect, reducing cell proliferation and increasing apoptosis. This suggests that *CDC45* contributes to LIHC progression by promoting cell growth.^50^ Moreover, *CDC45* was found associated with immune cell infiltration, the tumor microenvironment, and immune checkpoints, with lower *CDC45* expression correlating with a better response to immunotherapy in KIRC patients. Overall, *CDC45* shows potential as both a prognostic biomarker and a therapeutic target for both LIHC and KIRC, with lower expression suggesting improved sensitivity to immunotherapy.^51^ The *NDC80* exhibits important role in the kinetochore, a large protein machine that segregates chromosomes during cell division. The expression of *NDC80* in LIHC was analyzed in 47 paired LIHC and adjacent tissue samples using real-time reverse transcription polymerase chain reaction. In the LIHC cell line Sfx-7721, lentivirus-mediated silencing of *NDC80*, confirmed by PCR and western blotting, significantly reduced cell proliferation and colony formation, induced apoptosis, and caused cell cycle arrest at the S-phase. These findings suggest that elevated *NDC80* expression may contribute to LIHC development by inhibiting apoptosis and bypassing cell cycle arrest.^52^ Another study focused on *SPC25*, a component of the *NDC80* complex, analyzing its expression, survival prognosis, clinical features, and genetic changes across multiple cancer types, including LIHC and KIRC. *SPC25* was found to be highly expressed in most cancers, with associations to tumor mutational burden (TMB), microsatellite instability, and other clinical features.^53^

Lastly, the two hub genes *BIRC5* and *RRM2* play important role in regulation of gene expression. The *BIRC5* hub gene codes for the Survivin protein, which belongs to the inhibitor of apoptosis family. A recent pan cancer study found that *BIRC5* expression was dramatically upregulated in cancer tissue compared to normal tissue in 16 different cancer types suggesting it as a prognostic biomarker.^54^ High *BIRC5* levels were associated with increased mortality, particularly in kidney renal clear cell carcinoma (KIRC), liver hepatocellular carcinoma (LIHC), adrenocortical carcinoma, low-grade glioma, and lung adenocarcinoma. Variations in *BIRC5* expression were noted in relation to TP53 mutation status, tumor grade, and stage. *BIRC5* was found to interact with genes involved in apoptosis, cell division, cell cycle regulation, and cancer pathways, underscoring its oncogenic role in promoting cancer cell growth and development. Additionally, *BIRC5* expression was strongly correlated with immune cell infiltration, further influencing survival outcomes. These findings suggest that *BIRC5* is an independent unfavorable prognostic marker and a potential target for future cancer therapies.^55^, ^56^ On the other hand, *RRM2* (Ribonucleotide reductase M2) participates in the metabolism of nucleotides and catalyzes the change from nucleotides to deoxynucleotides. Thus, it regulates the dNTP pools for biosynthesis, repair, and replication of DNA.^54^ Pan-cancer mRNA expression profiling studies reported that *RRM2* is overexpressed in many cancers tissue compared to normal tissue.^57^ *RRM2* plays a critical function in tumor development and progression, and RRM2 overexpression is linked to worse patients’ outcomes in cancers^58^, ^59^. Suppression of RR enzyme activity could be used as a vital anticancer strategy, has been positively utilized in clinical control of multiple cancer malignancies^60^.

As discussed earlier, data mining revealed a significant correlation between *TOP2A* expression and clinical survival outcomes in KIRC and LIHC patients. Given its consistent upregulation and significant correlation with patient prognosis, TOP2A was selected for molecular docking studies. The docking results identified 14-Deoxy-11,12-didehydroandrographolide as a potential therapeutic compound, demonstrating strong binding affinity with TOP2A. Pharmacokinetic and toxicity assessments support its potential as a drug candidate. The well-known herb Andrographis paniculata yields a compound known as 14-Deoxy-11,12-didehydroandrographolide. To elucidate the clinical potential of *Andrographis paniculata* and support our predictions, we reviewed the relevant literature. Andrographolide, the active phytoconstituent derived from *Andrographis paniculata*, has been reported to alleviate various human ailments, including different forms of cancer. Molecular docking and network pharmacology have highlighted andrographolide’s anticancer activity, particularly against liver hepatocellular carcinoma (LIHC).^61^ Andrographolide exerts its anticancer effects by blocking key signaling pathways involved in cancer growth (JNK, NF-κB, and PI3K), reducing the activity of proteins that promote cancer cell division and metastasis (cyclins, CDKs, metalloproteinases, growth factors, and heat shock proteins), and increasing levels of tumor-suppressing proteins (p53 and p21), thereby inhibiting cancer cell growth, survival, spread, and angiogenesis.^62^ Additionally, andrographolide is widely used as a hepatoprotective medication, improving conditions such as alcoholic and non-alcoholic liver injuries and protecting the liver from hepatotoxic substances.^63^ Another study identified and tested non-andrographolide compounds from methanolic extracts of *Andrographis paniculata* leaves, revealing significant toxicity against various cancer cell lines, including HeLa, MCF7, BT549, 293, and A549, and demonstrated the extracts' impact on inhibiting cancer cell invasion and migration.^64^ Research on andrographolide derivatives has also shown promise, with compound 3g exhibiting strong cytotoxicity against HCT-116 colon cancer cells, and other derivatives like 3c showing significant effects on cell cycle progression and apoptosis.^65^, ^66^ The induction of cell cycle arrest is one of several mechanisms by which andrographolide exerts its anticancer effects.^67^ Furthermore, andrographolide has been shown to overcome tumor necrosis factor-related apoptosis-inducing ligand (TRAIL) resistance in renal cancer cells, effectively reducing cell viability, inhibiting proliferation, and enhancing apoptosis by restoring TRAIL signaling and upregulating the TRAIL receptor DR4, suggesting a promising therapeutic approach for renal cancer.^68^ These findings collectively underscore the potential of *Andrographis paniculata* as a therapeutic agent across a broad spectrum of malignancies.

## CRediT authorship contribution statement

**Maisha Tasneem:** Formal analysis, Methodology, Resources, Software, Writing – original draft. **Shipan Das Gupta:** Conceptualization, Investigation, Supervision, Writing – review & editing. **Md Jubair Ahmed Jony:** Writing – review & editing. **Maya Minkara:** Methodology, Validation, Writing – review & editing. **Rajib Kumar Dey:** Writing – review & editing. **Jannatul Ferdoush:** Funding acquisition, Investigation, Project administration, Resources, Supervision, Writing – review & editing.

## Declaration of competing interest

The authors declare that they have no known competing financial interests or personal relationships that could have appeared to influence the work reported in this paper.

## Data Availability

Data will be made available on request.
